# The effects of psychological risk factors at work on cognitive failures through the accident proneness

**DOI:** 10.1186/s40359-021-00669-5

**Published:** 2021-10-19

**Authors:** Milad Abbasi, Mohsen Falahati, Masoumeh Kaydani, Rohollah Fallah Madvari, Ahmad Mehri, Maryam Ghaljahi, Saeid Yazdanirad

**Affiliations:** 1 Occupational Health Engineering, Social Determinants of Health Research Center, Saveh University of Medical Sciences, Saveh, Iran; 2 Department of Occupational Health Engineering, Shoushtar Faculty of Medical Sciences, Shoushtar, Iran; 3 Department of Occupational Health Engineering, School of Public Health, Shahid Sadoughi University of Medical Sciences, Yazd, Iran; 4 Department of Occupational Health Engineering, School of Public Health, Hamedan University of Medical Sciences, Hamedan, Iran; 5 Department of Occupational Health Engineering, School of Public Health, Zabol University of Medical Sciences, Zabol, Iran; 6 Occupational Health Engineering, School of Health, Shahrekord University of Medical Sciences, Shahrekord, Iran; 7 Occupational Health Engineering, Modeling in Health Research Center, Shahrekord University of Medical Sciences, Shahrekord, Iran

**Keywords:** Psychosocial factor, Occupational factor, Cognitive failure, Accident proneness

## Abstract

**Background:**

Various agents such as psychosocial items and accident proneness can affect cognitive failures through different paths. The probable paths are the direct effects of workplace psychosocial items on cognitive failures and their indirect effects on cognitive failures through the mediator variable of accident proneness, which has not yet been studied by others. Thus, the present study aimed to investigate these paths.

**Methods:**

This cross-sectional study was conducted on 164 male employees of Karoon Sugar Company in 2018. The participants were asked to complete a background and demographic questionnaire, Broadbent cognitive failures scale, accident proneness questionnaire, and Copenhagen psychosocial questionnaire. Obtained data were analyzed and modeled using the statistical descriptive method, ANOVA, independent t-test, Pearson correlation test, and path analysis in the SPSS and AMOS software.

**Results:**

The results of the path analysis showed that, not only, some psychosocial risk items had a significant direct effect on cognitive failures, but also, they could affect cognitive failures through the accident proneness, indirectly. Work-family conflict and social support from supervisors by coefficients of 0.188 and – 0.187 had the highest direct effects, respectively. The highest indirect effects belonged to justice and respect, and work-family conflict by coefficients of - 0.220 and 0.199, respectively. The highest total effects were also related to the work-family conflict and justice and respect by coefficients of 0.387 and – 0.381, respectively.

**Conclusions:**

In total, our results showed that some psychological items could, directly and indirectly, increase cognitive failure through accident proneness.

## Background

Cognitive failures describe cognitive errors that occur in a simple task while it is expected that people perform them without any error [1]. These failures happen in four areas including attention, memory, perceptions, and motor function [2]. Indeed, this impairment is related to the disability and lapses at the attendance to a task that causes the errors in task execution [3]. This failure can be an indicator of the human information processing capacity and can affect task performance. Cognitive failure sometimes can lead to adverse consequences such as accidents at workplaces [4]. Barrett and Alexander demonstrated that there is a positive correlation between cognitive failure and accident occurrence. They stated that attention loss, distraction, and mental errors could cause accidents [5]. As well as, resulted by Wallace and Chen, individual cognitive failures are one of the reasons for increasing unsafe behaviors [6]. Hasanzadeh et al. also examined the relationship between occupational failure with unsafe behaviors and accidents at drivers. The results showed that cognitive failure is a substantial predictor of unsafe behaviors and accidents [7]. The results of a follow-up study showed that cognitive failure among workers in an army industry enhanced fall injury events and hospitalization [8]. The results of these studies indicated that cognitive failures play an important role in increasing workplace hazardous errors. There are some solutions to reduce cognitive failures. For example, Hsu et al. concluded that the use of workplaces flextime could decrease employees’ cognitive failures via increasing their perceived control [9]. However, there are various items such as psychosocial items, personal properties, and biological agents that negatively affect cognitive failure [10]. One group of the main agents is psychosocial items such as stress, justice, burnout, conflicts, supports, and demands. These workplace psychosocial items are results of interactions between work organization items and workers’ capacities, needs, and experiences [11]. Those can affect the workers’ performance. Stenfors et al. studied the relationship between psychosocial conditions at workplaces and cognitive complaints. The results showed that there are significant relationships between psychosocial conditions and cognitive complaints [12]. Day et al. also concluded that psychological stress could increase cognitive failures and accident occurrences at the workplaces [13]. Another important agent affecting cognitive performance is the individual differences such as personal properties and biological agents [14]. Wallace et al. showed that boredom proneness increases cognitive failures [15]. Unsworth et al. concluded that the individual difference in cognitive abilities including working memory, attention control, and retrospective and prospective memory substantially affect the everyday cognitive failures [16]. Accident proneness also is one of the individual differences that may influence cognitive failure. Indeed, the concept of accident proneness is applied to show that some persons experience more accident-related health problems compared to others [17]. It is different from injury vulnerability, as another effective agent on cognitive failure. Injury vulnerability elevates the risk of injury or illnesses while the accident proneness increases the probability of the accident occurrence by the people [18]. However, accident proneness overlaps with some personality traits such as conscientiousness and neuroticism. Elfering et al. concluded that there is an indirect path from conscientiousness to near-accidents via cognitive failure in action regulation [19]. In addition, the results of a study performed by Konen and Karbach showed that cognitive failures were significantly related to the personality domains of conscientiousness and neuroticism [20]. Moreover, the results of a systematic review indicated that personality traits of neuroticism, anxiety, and whilst hypomania have a significant effect on cognitive failures [10]. It is hypothesized that all workplace psychosocial items affect cognitive failures directly and indirectly through accident proneness as a mediator variable. Therefore, this study aimed to discover the various paths of the effect of psychosocial factors on cognitive failures. It is possible that only some psychosocial items influence cognitive failures, therefore, specifying the most effective factors is of most importance. Also, the difference of cognitive failure and accident proneness values were investigated in groups with various demographical characteristics.

## Methods

This descriptive-analytic study was conducted in Karoon Sugar Industry and Cultivation Company from July to November 2018. In this company, all employees were male (220 male workers). Sampling was done by taking the census and all staff expressed their oral consent to participate in the study voluntarily. Ethical approval for this study was obtained from the institutional research ethics committee of Saveh University of Medical Sciences (IR.SAVEHUMS.RWC.1398.014). The inclusion criterion of this study included males over 25 years old, five years of job experience, and the presence of any well-known mental illnesses in the participants. Based on these criteria, five workers were excluded and of the remaining voluntary workers for participating in the study, 164 subjects were selected based on inclusion criteria (participation rate was equal to 76%). At the first, a briefing session was held to familiarize workers with the objectives of the study and how to properly complete the questionnaires. In the next step, the subjects were asked to fill out the questionnaires including demographic and background questionnaire, cognitive failure questionnaire, accident proneness questionnaire, and Copenhagen psychosocial questionnaire, carefully. The questionnaires are described as follows.

### Cognitive failures questionnaire (CFQ)

This questionnaire is based on Broadbent’s cognitive failure theory [21]. This questionnaire is to measuring cognitive failures in four areas including memory, memory name, distractibility, social blunders. This questionnaire considers the various dimensions of cognition, cognition properties, and the layers in which cognitive failures occur. CFQ consists of 25 questions that cover all four types of failures. In a study conducted by Allahyari et al., the validity, internal consistency, and repeatability of this tool were determined. The results showed that the content validity index (CVI) and alpha Cronbach coefficient were 0.7 and 0.96, respectively [22]. The scoring is based on a Likert scale from zero (never) to four (very high) thus, the total score of CFQ can range from zero to 100. A higher score indicates cognitive failure.

### Accident proneness questionnaire

This tool contains nine dimensions including personality traits, workplace harmful agents, safety culture, safety attitude, occupational stress, musculoskeletal disorders, organizational interest, degree of risk-taking, and individual items. Each dimension has a loading factor and each question has a specific impression coefficient. To achieve the total score, the responses were multiplied by their impression coefficient. Then, the obtained scores for each dimension were added together and multiplied by the loading factor. The sum of all dimensions shows the total score of the accident proneness. The higher score indicates the greater individual accident proneness. The scores were categorized as scores lower than 82.5 as acceptable or low, 82.6–114.5 as moderate, 114.6–148.5 as high, and higher than 148.5 as very high. This questionnaire was developed and validated by Karimi et al. and is a reliable and valid instrument for screening accident-prone people in the industries. Its alpha Cronbach coefficient was 0.86 and the results of the confirmatory factor analysis demonstrated that the presented model was fitted with real data [23]. This questionnaire is a proper tool for measuring accident proneness and evaluates the social, individual, organizational, and occupational items.

### Copenhagen psychosocial questionnaire (COPSOQ)

Copenhagen psychosocial questionnaire (COPSOQ) is an instrument that is extensively used for the assessment of psychological items at work. In the present study, a short version of this questionnaire was used that has been validated by Aminian et al. The construct validity of the Farsi COPSOQ was confirmed by the factor analysis. As well as, the range of alpha Cronbach was between 0.75 and 0.89 [24]. This tool has 32 questions and 16 dimensions including the meaning of work, quality of leadership, influence at work, role clarity, justice and respect, rewards, trust, predictability, self-rated health, burnout, stress, social support from supervisors, work-family conflict, emotional demands, commitment to the workplace, and offensive behavior. To complete the questionnaire, the subjects answered each question using a five-point Likert scale from zero (always) to four (never).

### Statistical analysis

At the first, data were categorized and described using descriptive statistical methods. One-way ANOVA and independent t-test were used to compare the mean values of dependent variables between different groups. Principal component analysis (PCA) was also conducted to reduce the dimensionality of the data set. Moreover, the Pearson correlation test was used to determine the correlation between the variables of cognitive failures and accident proneness with psychosocial items. Therefore, it is hypothesized that psychosocial factors can affect cognitive failures through accident proneness as a mediator variable. Thus, the path analysis was applied to acknowledge if psychosocial and individual items have direct and indirect effects on cognitive failures. The analysis was performed in SPSS and Amos software environments.

## Results

The results showed that 56 (34.1%) and 108 (65.9%) subjects of the participants were single and married, respectively. Based on the type of employment, 24 (14.6%) subjects were domestic subcontractors, 105 (64.1%) subjects had casual work contracts, and 35 (21.3%) remaining subjects have full-time permanent contracts. The participants were working at three parts including steam furnace (32 (19.5%) subjects), mill (56 (34.1%) subjects), and raw materials and sugar filtration (76 (46.4%)) and in three work shifts including morning (55 (33.5%) subjects), evening (52 (31.8%) subjects), and night (57 (33.5%) subjects). Of the participants, 57 (34.8%) individuals were with education degrees less than a diploma, 78 (47.6%) individuals with diplomas, and 29 (17.6%) individuals with bachelor’s and higher degrees. As well as, 29 (17.6%) people had a second job. The mean and standard deviation of the age and work experience of the participants were 39.15±6.89 and 13.34±6.22, respectively. The mean and standard deviation of the cognitive failure (CF) and accident proneness (AP) were equal to 66.43 ±16.96 and 124.17±10.74. The mean, standard deviation, maximum Z score, and minimum Z score values of the quantitative variables are presented in Table 1.

In Table 2, the mean of CF and AP among different groups of the understudied qualitative variables in different working departments are presented. This table shows the results of one-way ANOVA and independent t-test. Based on the results, the mean values of accident proneness and cognitive failure were significantly higher in people with the second job, married, with casual work contract and domestic subcontractor, with night shift work, and with low education level.

**Table 1:** Descriptive statistics of studied variables

Variables	Mean	Standard deviation	Z score	
			Minimum	Maximum
Age	39.15	6.89	− 2.24	2.57
Work experience	13.34	6.22	− 1.98	2.25
Accident proneness	124.17	10.74	− 2.20	2.72
Cognitive failure				
Memory	21.45	5.68	− 2.44	2.11
Memory name	5.50	1.83	− 3.14	1.87
Distractibility	21.00	5.81	− 2.36	2.09
Blunders	18.70	4.90	− 2.40	2.04
Total	66.43	16.96	− 2.42	2.44
Psychosocial items				
Quality of leadership	3.29	1.89	− 1.90	2.28
Supervisors social support	4.32	1.92	− 1.78	2.45
Rewards	4.87	2.38	− 2.36	1.72
Justice and respect	4.34	2.45	− 1.94	2.36
Trust	3.26	1.72	− 2.18	2.41
Predictability	3.35	1.88	− 1.95	2.70
Burnout	3.58	2.13	− 2.44	1.99
Stress	3.71	2.01	− 1.98	1.96
Work-family conflict	3.53	2.33	− 2.15	2.36
Meaning of work	1.17	0.98	− 1.75	2.97
Influence at work	2.38	1.43	− 1.95	3.17
Role clarity	2.70	2.19	− 1.89	2.77
Offensive behavior	6.84	1.25	− 4.38	1.25
Emotional demands	2.09	1.21	− 1.76	1.84
Commitment to workplace	0.83	0.73	− 1.81	3.60
Self-rated health	1.88	1.24	− 2.39	2.19

**Table 2:** The mean of CF and AP among different groups of the understudied qualitative variables

Variable	Steam furnace department		Mill department		Sugar filtration department		AP			CF		
							*P* value	95% Confidence Interval for Mean		*P* value	95% Confidence Interval for Mean	
	AP	CF	AP	CF	AP	CF		Lower Bound	Upper Bound		Lower Bound	Upper Bound
Second job												
Have	128.08	59.44	127.59	69.50	133.21	80.57	0.001^†^	3.35 ^a^	11.74 ^a^	0.015 ^†^	1.44 ^a^	14.88 ^a^
Do not have	113.68	44.40	127.52	71.47	123.52	69.04						
Marital												
Single	115.18	49.47	124.59	64.80	121.58	67.83	0.020^†^	− 7.38^a^	− 0.48^a^	0.060^†^	− 10.45^a^	0.43^a^
Married	115.46	45.33	129.30	74.91	127.02	72.71						
Type of employment												
Domestic subcontractor	115.57	44.66	133.68	79.37	133.52	81.07	0.001^‡^	122.51	125.82	0.001^‡^	63.79	68.99
Casual work contract	116.71	49.36	126.56	69.69	124.61	70.92						
Permanent contract	113.07	41.66	126.39	69.86	118.86	60.63						
Type of shiftwork												
Morning	113.63	43.70	111.57	46.85	116.62	43.01	0.001^‡^	122.51	125.82	0.001^‡^	63.79	68.99
Evening	127.05	60.55	124.45	62.73	122.16	69.01						
Night	122.17	74.01	132.18	79.82	138.31	91.77						
Education level												
> diploma	108.97	40.20	120.77	60.01	119.65	64.13	0.001^‡^	122.51	125.82	0.044^‡^	63.79	68.99
Diploma	128.62	59.20	128.89	74.31	122.88	68.05						
Bachelor <	123.88	60.01	131.79	76.02	139.90	89.60						

^†^Independent t test

^‡^Variance analysis test

^a^95% confidence interval of the difference

The Kaiser–Meyer–Olkin measure of sampling adequacy obtained for PCA was equal to 0.894. Therefore, PCA may be useful with the data. Table 3 reports the results of the component matrix. Based on the results of PCA, the three principal components were identified, which had eigenvalues greater than 1 and explained more than 60% of the variation in the data. The first principal component increases with increasing supervisor’s social support, role clarity, justice and respect, commitment to the workplace, meaning of work, quality of leadership, and rewards. Additionally, this principal component decreases with increasing work-family conflict, stress, and burnout. The results showed that the second principal component was positively associated with trust, predictability, and self-rated health. It was negatively associated with offensive behavior. Moreover, the third principal component was positively associated with emotional demands and influence at work.

The results of the Pearson test, presented in Table 4, indicated that cognitive failure has significantly positive relationships with age, work experience, burnout, stress, work-family conflict, and influence at work. However, quality of leadership, social support from supervisors, rewards, justice and respect, meaning of work, role clarity, offensive behavior, commitment to the workplace, and self-rated health had significantly negative relationships with cognitive failure. As well, the accident proneness showed significantly positive relationships with the variables of age, work experience, burnout, stress, work-family conflict, and influence at work. However, the relationships of the accident proneness with the variables of the quality of leadership, supervisor’s social support, rewards, justice and respect, burnout, stress, and influence at work were significantly negative. Accident proneness was significantly associated with age, experience, burnout, stress, work-family conflict, and influence at work. More detailed results are presented in Table 4.

**Table 3:** Component matrix

Variable	Component			Eigen value of variables
	1	2	3	
Supervisor’s social support	0.870	–	–	1.586
Work-family conflict	− 0.829	–	–	0.996
Role clarity	0.810	− 0.112	0.146	0.926
stress	− 0.774	–	–	0.831
Justice and respect	0.763	–	–	0.690
Commitment to the workplace	0.756	–	–	0.573
Meaning of work	0.725	–	0.243	0.523
Quality of leadership	0.680	0.102	–	0.489
Rewards	0.672	0.341	–	0.424
Burnout	− 0.621	− 0.268	0.181	0.375
Trust	–	0.706	–	0.350
Predictability	0.113	0.574	− 0.140	0.318
Offensive behavior	0.219	− 0.568	0.325	0.246
Self-rated health	0.283	0.380	–	0.235
Emotional demands	–	0.173	0.834	0.160
Influence at work	− 0.218	0.240	0.784	0.054

Extraction Method: Principal Component Analysis

3 components extracted

**Table 4:** The relationship of cognitive failure and accident proneness with quantitative variables

Variables	Cognitive failure		Accident proneness	
	r	*P* value	r	*P* value
Age	0.198*	0.011	0.248**	0.001
Work experience	0.160*	0.041	0.192*	0.014
Psychosocial items				
Quality of leadership	− 0.616**	0.001	− 0.584**	0.001
Social support from supervisors	− 0.853**	0.001	− 0.731**	0.001
Rewards	− 0.568**	0.001	− 0.471**	0.001
Justice and respect	− 0.759**	0.001	− 0.701**	0.001
Trust	0.111	0.156	0.149	0.056
Predictability	− 0.063	0.424	− 0.017	0.831
Burnout	0.541**	0.001	0.479**	0.001
Stress	0.735**	0.001	0.664**	0.001
Work-family conflict	0.823**	0.001	0.723**	0.001
Meaning of work	− 0.690**	0.001	− 0.624**	0.001
Influence at work	0.199*	0.011	0.207**	0.008
Role clarity	− 0.776**	0.001	− 0.676**	0.001
Offensive behavior	− 0.246**	0.002	− 0.287**	0.001
Emotional demands	− 0.055	0.486	− 0.067	0.394
Commitment to the workplace	− 0.734**	0.001	− 0.670**	0.001
Self-rated health	− 0.180*	0.022	− 0.228**	0.003

*Significant at error level of 0.05

**Significant at error level of 0.01

**Fig. 1:**
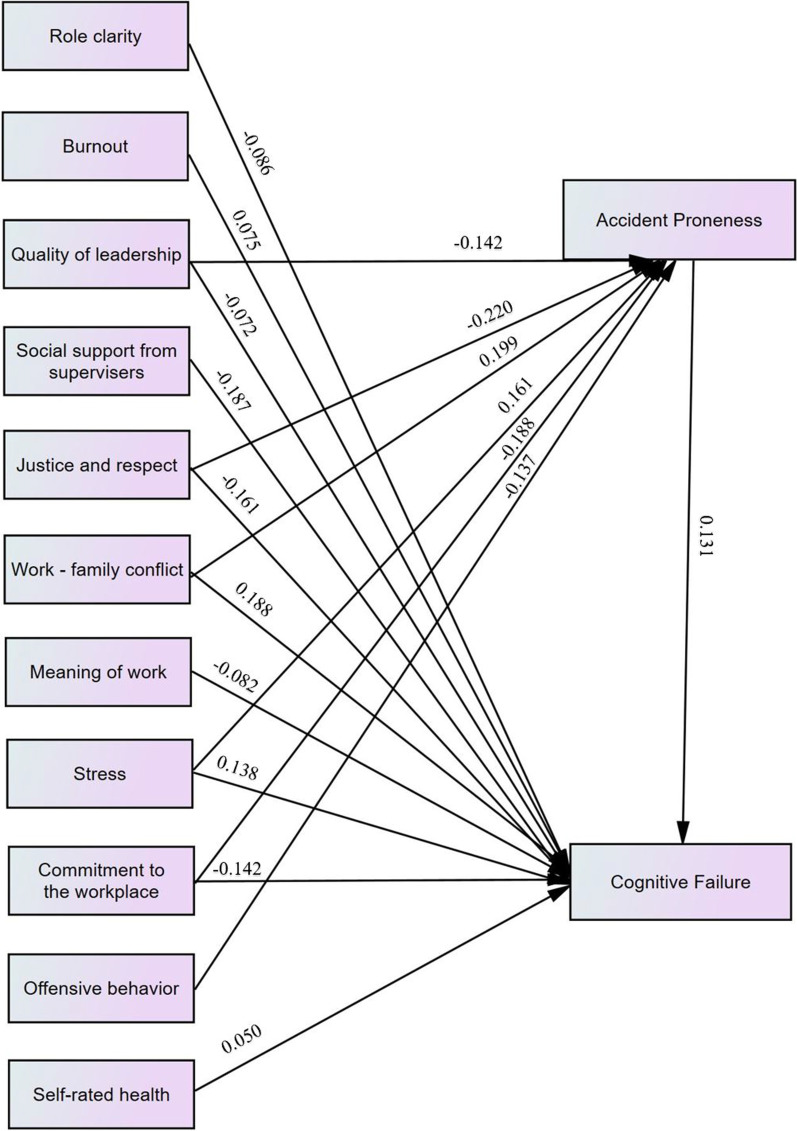
Path analysis of studied variables on cognitive failure

To determine the direct and indirect effect of psychosocial items on cognitive failure, the items were included in the path analysis model. In this analysis, the accident proneness was considered as a mediator variable. The results of the path analysis are presented in Table 5; Fig. 1. Based on the results, the variables of rewards related to the first principal component, trust and predictability related to the second principal component, and influence at work and emotional demands related to the third principal component could not significantly affect the cognitive failure from both direct and indirect paths. Therefore, nine items of first factors and two items of second factors remained in the model. Given the aim of the study, the items were not grouped based on the results of PCA and the effect of each variable on the accident proneness and cognitive failure were separately investigated in the final model. It should be noted that several models were drawn using AMOS software and the goodness-of-fit indices related to the model with three components were not in the optimal ranges while entering all related variables to the model separately led to the confirmation of these indices. It may be due to that the collinearity between three components, which can make a negative effect on the accuracy of the model.

The results of this showed that not only some psychosocial items have a significant direct effect on cognitive failures, but also, they could affect cognitive failures through the accident proneness, indirectly. However, the results showed that the variables of quality of leadership, social support from supervisors, justice and respect, burnout, stress, work-family conflict, meaning of work, work clarify, self-rated health, and commitment to the workplace had significant direct effects on the cognitive failure. Social support from supervisors and work-family conflict with the standardized direct effect coefficients of – 0.187 and 0.188 were the most effective negative and positive items, respectively. Moreover, the variables of quality of leadership, justice and respect, stress, work-family conflict, offensive behavior, and commitment to the workplace could indirectly affect cognitive failures through the accident proneness. The highest indirect effects belonged to justice and respect and work-family conflict with the coefficients of – 0.220 and 0.199, respectively. In addition, the greatest total effects were also related to the variables of work-family conflict and justice and respect with the coefficients of 0.387 and – 0.381, respectively. Based on the results, elevated accident proneness, burnout, stress, and work-family conflict can trigger increased cognitive failure. In contrast, diminished quality of leadership, social support from supervisors, justice and respect, meaning of work, role clarity, offensive behavior, commitment to the workplace, and self-rated health can lead to increased cognitive failure.

**Table 5:** The direct, indirect and total effect of studied variables on cognitive failure

Independent variables	Effects		
	Direct effect	Indirect effect	Total effect (direct+ indirect)
Accident proneness	0.131	–	0.131
Quality of leadership	− 0.072	− 0.142	− 0.214
Social support from supervisors	− 0.187	–	− 0.187
Justice and respect	− 0.161	− 0.220	− 0.381
Rewards	–	–	–
Trust	–	–	–
Predictability	–	–	–
Burnout	0.060	–	0.060
Stress	0.138	0.161	0.299
Work-family conflict	0.188	0.199	0.387
Meaning of work	− 0.082	–	− 0.082
Influence at work	–	–	–
Role clarity	− 0.086	–	− 0.086
Offensive behavior	–	− 0.137	− 0.137
Emotional demands	–	–	–
Commitment to the workplace	− 0.142	− 0.188	− 0.330
Self-rated health	− 0.050	–	− 0.050

## Discussion

The two probable paths were considered as the direct effects of workplace psychosocial items on cognitive failures and their indirect effects on cognitive failures through the mediator variable of accident proneness. Therefore, it was hypothesized that all workplace psychosocial items affect cognitive failures directly and indirectly through accident proneness as a mediator variable. However, our results suggested that only some of the psychosocial items were effective on cognitive failures through the mentioned paths. Considering our findings, the significant items with a high coefficient effect were identified. In addition, the relation of background and demographic variables with cognitive failures and accident proneness has been investigated. However, more studies are needed to confirm these results and trends.

Based on our results, in terms of demographical characteristics, the mean values of cognitive failure and accident proneness in the subjects with a second job were significantly higher than those without a second job. The people who have a second job undergo more responsibility that in turn it may be associated with more fatigue and burnout. This fatigue possesses negative outcomes including impaired cognitive performance, changed coping styles, decreased productivity, and health problems such as depression and cardiovascular disease [25, 26]. On the other hand, having a second job and in turn, increased responsibility and roles can also trigger the work-family conflict. Moreover, married individuals compared to single persons probably have more family-work conflict. Cognitive failure is affected by disturbing work–family, family, and work conflict. In addition, our results proposed that the workers with casual work contracts and domestic subcontractors experienced higher cognitive failure and accident proneness compared to subjects who were employed permanently. It may be because those people who are in tension, feel fear for losing the job, and do not have job security. Lieberman et al. concluded that stress and tension adversely affect cognitive performance and mood [27]. As well as, the workers with casual work contracts and domestic subcontractors may be less committed to the organization and perform their responsibility with lower attention and in turn, more cognitive failure. Based on the obtained results, the mean values of cognitive failure and accident proneness were higher in the night workers. Ozdemir et al. investigated the effect of shift work on cognitive functions. They showed that the scores of verbal memory, attention – concentration, immediate memory, and total learning were lower for the shift workers [28]. The results of a systematic review and meta-analysis show that the shift workers have a trend toward an increased risk of chronic sleep disturbances such as insomnia symptoms (risk ratio 1.16, 95% Confidence interval 0.97–1.38) [29]. As well, the results of another systematic review on sleep in the offshore petroleum industry revealed that the shift workers report more sleep problems [30]. Based on the theoretical model presented by Kecklund and Axelsson, shift work can disturb the sleep process through the circadian rhythm disruption and cause cognitive impairments such as variability in attention and lapses, poorer working and short-term memory, worse executive functioning, and poorer emotion regulation [31]. In addition, it can probably create risky behaviors and psychosocial stress [31]. The finding of these studies declares the statistical and important role of the shift work on cognitive failure and even high-risk behavior. In our study, the workers of the steam furnace department experienced lower cognitive failure and accident proneness compared to the workers of the other two departments. Perhaps, the environmental and job-specific characteristics in these departments can justify this result. Elmenhorst et al. state that noise exposure can adversely affect cognitive performance [32]. In the present study, the education level had a significant protective effect on cognitive failure and accident proneness. Schneeweis et al. observed that education possesses a protective effect on cognitive decline. They stated that a 1-year education would enhance the memory score approximately four decades later by 0.2 [33]. However, these results require more investigations in the next studies.

Considering the results of PCA, three principal components were identified but most of the psychological factors were in the first component. Moreover, of drawn pre-models, the goodness-of-fit indices of the model with these three components were not favorable while the goodness-of-fit indices for the model involving all psychological factors separately were observed as acceptable and reliable. Moreover, the accuracy of the model with three groups of variables, as the results of PCA, was questionable because of the collinearity between the three components. For this reason, the items were not classified based on the results of PCA and the effect of each variable on the accident proneness and cognitive failure were separately investigated (Fig. 1). The interpretation of our results could go in the direction that, of the ten items, one item was related to the first principal component (rewards). Moreover, two items of the four items related to the second principal component (trust and predictability), and all items related to the third principal component (influence at work and emotional demands) have no significant impact on the cognitive failure from both direct and indirect paths while the results of Pearson correlation test showed that rewards and influence at work were significantly associated with cognitive failure. It may be argued that the simultaneous effect of all items is tested in the path analysis and the results indicated that these two variables alongside others do not affect cognitive failure. Therefore, it seems that conducting path analysis based on the classifications of the variables into three components is not optimal.

Our results proposed that not all the workplace psychosocial factors can affect cognitive failures directly and indirectly and only some of them were effective on cognitive failure. Of the remaining variables, ten workplace psychosocial items had a direct effect on cognitive failure but only six of them possessed indirect effects. Hence, it is probable that these items mostly affect cognitive failure through the direct path rather than the indirect path. Considering our results, the variables of quality of leadership, social support from supervisors, justice and respect, burnout, stress, work-family conflict, meaning of work, work clarity, self-rated health, and commitment to the workplace have significant direct effects on the cognitive failure. In addition, accident proneness was one of the most effective items on cognitive failure. Previous studies have investigated the effect of cognitive failures on accident proneness. For example, Andrea et al. concluded that distressed individuals tend to commit more cognitive failure, in turn, more occupational accidents [13]. In the present study, an inverse relationship was also found between accident proneness and cognitive failure. Based on our findings, work-family conflict and social support from supervisors with the standardized direct effect coefficients of 0.188 and − 0.187 were the most effective negative and positive items, respectively. Based on the results of PCA analysis, social support from supervisors and work-family conflict with coefficients of 0.870 and - 0.829 had the highest association with cognitive failure. Nakata et al. studied the effect of job stress and social support from supervisors on the prevalence of insomnia in a population of Japanese daytime workers. They declare that some of the psychological items such as interpersonal conflicts with employees, job satisfaction, and social support modestly increase the risk of insomnia [34]. The results of a study performed by Abu-Alrub also showed that the reduced social support in the workplaces decreased job performance and increased job stress [35]. Therefore, the item of social support from supervisors may affect cognitive performance through stress and insomnia. In the present study also, stress was an effective item on cognitive failure. Work-family conflict was another significant item as well. Lapierre et al. concluded that family interference with work can positively be associated with workplace cognitive failure [36]. The results of a study performed by Arshadi et al. indicated that work-family conflict can significantly affect the overall health, workplace cognitive failure, and marital satisfaction and is an important issue in organizational behavior [37]. Hence, it is possible that the items of social support from supervisors and work-family conflict are substantial agents affecting cognitive failures. In addition, our results propose that variables of quality of leadership, justice and respect, stress, work-family conflict, offensive behavior, and commitment to the workplace can indirectly affect cognitive failures through accident proneness. In the present study, the highest indirect effects belonged to justice and respect and work-family conflict with the coefficients of − 0.220 and 0.199, respectively. In addition, the greatest total effects were also related to the variables of work-family conflict, and justice and respect with the coefficients of 0.387 and – 0.381, respectively. Gyeke and Habatollahi concluded that the level of perceived justice in an organization, especially relational justice, might affect job satisfaction, safe behaviors, and accidents [30]. Based on the social exchange theory, workers with positive perceptions of fairness, by acting safely, probably express their commitment and interest to their organization [38]. Additionally, Bridger et al., concluded that several psychosocial stressors, including work-family conflict, effort-reward imbalance, role conflict, and dissatisfaction with the physical work environment can be associated with the stress and lead to accidents in this way [39]. Hence, it seems that two items of justice and respect and work-family conflict are important agents. It is suggested that those and other identified items are considered and controlled in the workplace to reduce cognitive failure and increase performance. The manager can focus on these agents based on their effect coefficient and priority. However, more studies are needed to confirm these trends.

As one of the limitations in this study, all participants were male and the stated paths were not studied on the female workers. Therefore, it is recommended that the stated items and paths are also investigated in female employees in the next studies. Moreover, there was a likelihood of self-report bias due to the use of the questionnaire tools. Also, possibly electroencephalography examination was not conducted for the diagnosis of cognitive failures in the present study. However, it was tried to decrease this bias through surveillance on questionnaire complementation and presentation of necessary explanations. The clinical and physiological tools can be applied in the next studies. Additionally, in our study, due to the limited sample participating in the study, the internal relationships of psychosocial items in the model were not investigated. Hence, it is proposed that another study with a high number of samples is performed to evaluate these relationships among variables. Moreover, the specific environmental and occupational conditions in the study sample have constrained the originality of the results. Therefore, further studies in larger samples and different working conditions are recommended to cover the limitations of our study.

## Conclusion

In total, our results proposed that elevated burnout, stress, and work-family conflict and diminished quality of leadership, social support from supervisors, justice and respect, meaning of work, role clarity, commitment to the workplace, and self-rated health can directly trigger increase cognitive failure. Moreover, considering our findings, increasing stress and work-family conflict and decreasing quality of leadership, justice and respect, offensive behavior, and commitment to the workplace could indirectly impress on cognitive failures through the accident proneness. Therefore, the finding of our study may be helpful to decrease the cognitive failures in the workplaces and consequently to increase the performance and decrease the accident in a population with similar working conditions and demographic characteristics.

## Data Availability

The datasets used and/or analyzed during the current study are available from the corresponding author on reasonable request.

## Abbreviations

CFQ: cognitive failures questionnaire
CVI: content validity index
COPSOQ: Copenhagen psychosocial questionnaire
